# GC–MS Analysis, Molecular Docking and Pharmacokinetic Properties of Phytocompounds from *Solanum torvum* Unripe Fruits and Its Effect on Breast Cancer Target Protein

**DOI:** 10.1007/s12010-021-03698-3

**Published:** 2021-10-13

**Authors:** R. Saravanan, K. Raja, D. Shanthi

**Affiliations:** Post Graduate and Research Department of Zoology, Dr. Ambedkar Government Arts College, Vyasarpadi, Chennai, 600 039 Tamil Nadu India

**Keywords:** *Solanum torvum*, GC–MS, Molecular docking, SwissADME, Breast cancer

## Abstract

This study was designed to identify phytocompounds from the aqueous extract of *Solanum torvum* unripe fruits using GC–MS analysis against breast cancer. For this, the identified phytocompounds were subjected to perform molecular docking studies to find the effects on breast cancer target protein. Pharmacokinetic properties were also tested for the identified phytocompounds to evaluate the ADMET properties. Molecular docking studies were done using docking software PyRx, and pharmacokinetic properties of phytocompounds were evaluated using SwissADME. From the results, ten best compounds were identified from GC–MS analysis against breast cancer target protein. Of which, three compounds showed very good binding affinity with breast cancer target protein. They are ergost-25-ene-3,6-dione,5,12-dihydroxy-,(5.alpha.,12.beta.) (− 7.3 kcal/mol), aspidospermidin-17-ol,1-acetyl-16-methoxy (− 6.7 kcal/mol) and 2-(3,4-dichlorophenyl)-4-[[2-[1-methyl-2-pyrrolidinyl]ethyl amino]-6-[trichloromethyl]-s-triazine (− 6.7 kcal/mol). Further, docking study was performed for the synthetic drug doxorubicin to compare the efficiency of phytocompounds. The binding affinity of ergost-25-ene-3,6-dione,5,12-dihydroxy-,(5.alpha.,12.beta.) is higher than the synthetic drug doxorubicin (− 7.2 kcal/mol), and the binding affinity of other compounds is also very near to the drug. Hence, the present study concludes that the phytocompounds from the aqueous extract of *Solanum torvum* unripe fruits have the potential ability to treat breast cancer.

## Introduction

Breast cancer is one of the major problems for most women worldwide. About 10% of breast cancer occurrences are due to gene mutations which are inherited [1]. The pharmacologic medications, some of which are under present use, are taxanes, doxorubicin, epothilones, vincristine, camptothecin, tamoxifen and orraloxifene to prevent breast cancer but are at high risk of developing side effects. These anticancer agents cause disruption in the normal physiological functioning of vital organs resulting in neuropathy, nephrotoxicity and chemotherapy resistance [2]. Epidemiological studies suggest that antioxidant supplements might reduce the risk of breast cancer recurrence or mortality and lower the incidence of cancers in women [3].

Hereditary mutations in the germ cell may also be a related to cancers of the breast and ovary [4, 5]. The occurrence of mutations in BRCA1 gene is a risk factor for the development of breast cancer [6, 7]. The BRCA1 protein plays a role in the differentiation of breast epithelial cells. BRCA1 is a tumor suppressor gene. The loss of BRCA1 function results in reduced acini formation and an accumulation of less differentiated cells with different proliferation properties [8].

The expression of a functional BRCA1 protein plays an important role in breast carcinogenesis [9]. BRCA1 has been mapped to long arm of chromosome 17 at 17q21. It encodes a nuclear protein of 1863 amino acids. The gene contains 24 exons, its coding region start from the middle of exon that ranges over 80 kb. It regulates transcriptional activation, maintenance of genomic integrity and stability, sex chromosome inactivation, ubiquitination, DNA repair, apoptosis, cell-cycle checkpoint control and chromosomal remodeling [10, 11]. The functional role of estrogen in breast cancer etiology and the potential integrative role between BRCA1 and the hormone synthesis are under-investigated in BRCA1 [12].

BRCA1 is identified as p53 interacting protein [13]. It interacts with RAD 51, a protein which is a major component in DNA repairing mechanism and DNA recombination. BRAC1 forms complex, which will further initiate the repairing of double-strand breaks [14, 15]. BRAC1-associated genome surveillance complex (BASC) comprises tumor suppressor gene involved in the DNA repairing process [16, 17]. Damage that occurs to BRCA1 by chance prevents the protein from DNA repair process and thereby increases the risk of developing tumor [18, 19].

Naturally occurring compounds from herbal resources with medicinal value have provided innumerable chemotherapeutics and will sustain to be an important component of drug discovery for futuristic approach [20]. There are growing evidences in the treatment of cancers by using plant origin-based phytocompounds with chemopreventive properties [21, 22]. Pharmacological studies of *Solanum torvum* fruits revealed antiviral, immunosecretory, antioxidant, analgesic, anti-inflammatory and anti-ulcerogenic activities [23–25].

Molecular docking approaches are used in modern drug design to understand drug-receptor interaction [26, 27]. Computational techniques strongly support and help the design of novel, more potent inhibitors by revealing the mechanism of drug-receptor interaction. The Swiss ADME web tool is a freely available software used to predict the physicochemical properties, absorption, distribution, metabolism, elimination and pharmacokinetic properties of molecules, which are key attempts for further clinical trials [28, 29]. The goal of ligand–protein docking is to predict the predominant binding model of a ligand with a protein of known three-dimensional structure [30].

The main objective of this study was to identify potential phytocompounds (ligands) from the aqueous extract of *Solanum torvum* unripe fruits for breast cancer susceptibility protein belonging to BRCA1 gene. This would be a future explorer for designing a promising novel drug, by reducing the time span of drug discovery, and could further be explored as possible therapeutic intervention for breast cancer.

## Materials and Methods

### Collection and Identification of Plant Material

The unripe fruits of Solanum torvum (S. torvum) were collected from in and around Kancheepuram District (12.8185°N, 79.6947°E), Tamil Nadu during the month of May to June 2018. The plant specimen was authenticated (Registration No.: PARC/2018/3855) by Dr. P. Jayaraman, Director, Plant Anatomy Research Center, Tambaram, Chennai.

### Processing and Preservation of Plant Material

The unripe fruits of *Solanum torvum* (*S. torvum*) were washed with running tap water and rinsed in distilled water. The unripe fruits were chopped into small pieces and shade dried for 2 weeks for complete dryness. The dried unripe fruits were grinded to a fine powder using a mechanical grinder. The powdered material was stored in airtight containers for further use.

### Preparation of Aqueous Extract (Maceration by Cold Extraction Method)

About 50 g of the dried fruit samples was separately weighed, soaked and dissolved in 600 ml of distilled water in a 1000-ml conical flask and placed for cold maceration for about 7 days at normal room temperature. The flask was tightly plugged with absorbent cotton and aluminum foil and was stirred periodically using a rotary shaker for 24 h. The extract was filtered using Whatman no. 1 filter paper. The final yield (15 g) of filtered extracts in the form of concentrated paste was used for further study.

### Gas Chromatography-Mass Spectrometry (GC–MS) Analysis

GC–MS analysis of aqueous extract of *S. torvum* unripe fruit was performed using GC–MS (Model: GC MS—QP 2010, Shimadzu, Japan) equipped with a VF-5 ms fused silica capillary column of 30 m length, 0.25 mm diameter and 0.25 µm film thickness. For GC–MS analysis, electron ionization system with ionization energy of 70 eV was used. The carrier gas used was helium (99.9%), at a constant flow rate of 1.2 ml/min. Injector and mass transfer line temperature were set to 280 °C and 255 °C respectively. The oven temperature was set from 50 to 250 °C at 10 °C/min for 5 min and finally raised to 300 °C for 10 min. Two microliters of the sample was injected in a split mode with a scan range of 50–1000 m/z. The total running time of GC–MS was 49 min. The relative percentage amount of each component was calculated by comparing its average peak area normalization value.

## In Silico Studies

### Ligand Selection

From the GC–MS analysis of the aqueous extract of *S. torvum* unripe fruits, 236 compounds were identified. All the compounds were analyzed. The 3D structure of all the compounds was retrieved from the PubChem database (https://pubchem.ncbi.nlm.nih.gov/) and used in this study.

### Selection of Target Protein

The target protein breast cancer type-1 susceptibility protein that belongs to BRCA1 gene was found from the literature. The 3D structure of this target protein was retrieved from PDB database (https://www.rcsb.org). The UniProt ID of this target protein was taken from the Uniprot database (https://www.uniprot.org/). The 3D structure of target protein breast cancer type 1 susceptibility protein for breast cancer was obtained from PDB database, and its PDB ID is 1JNX, and the UniProt ID of this target protein is P38398.

### Docking Studies

Docking studies for the target protein, breast cancer type-1 susceptibility protein, BRCA1 and phytocompounds (ligands) of *S. torvum* fruit were performed using PyRx software [31]. All the ligands were prepared using Open Babel option in the PyRx, and the target protein was prepared using Discovery Studio 2021. The results were also analyzed using Discovery Studio 2021.

### Pharmacokinetic Properties

ADMET properties were tested for the best interacted phytocompounds from the aqueous extract of *S. torvum* unripe fruits using SwissADME [32]. Lipophilicity (XLogP_3_), topological polar surface area (TPSA), solubility and hydrophobicity (Log S), carbon fraction sp3 (saturation carbons in sp3 hybridization) and rotatable bonds (flexibility) Lipinski rule, blood–brain barrier (BBB), human intestinal absorption (HIA), P-glycoprotein (PGP), cytochrome P450 inhibitor isoenzymes and skin permeation parameters were evaluated for all the compounds.

## Results

### GC–MS Analysis

**Fig. 1:**
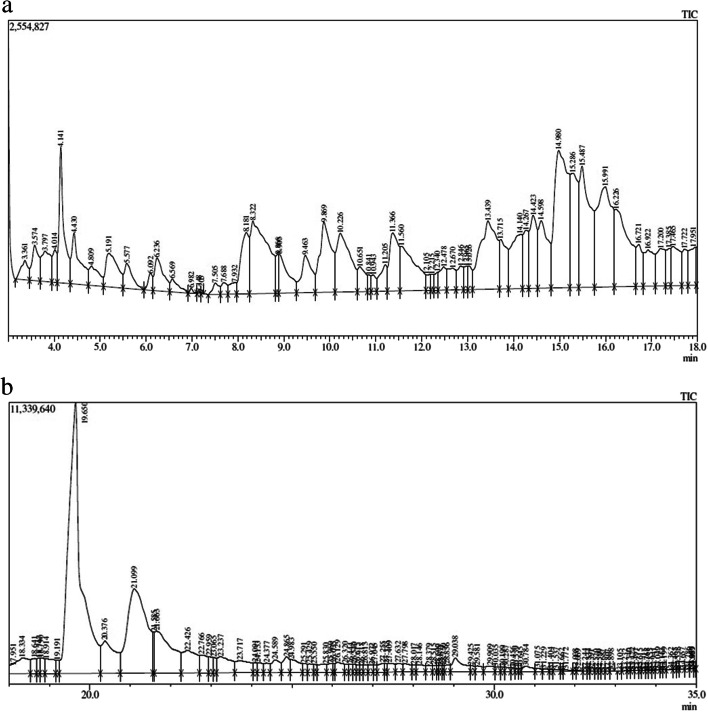
**a** The GC–MS chromatogram of the aqueous extract of *S. torvum* unripe fruits. **b** The GC–MS chromatogram of the aqueous extract of *S. torvum* unripe fruits

The chemical spectrum profile of *S. torvum* unripe fruit extract by GC–MS data was compared with the known compounds stored in the NIST library attached to the GC–MS. A total of 236 chemical structures were identified. Of which, 165 compounds were interacted with the target protein. Among these compounds, ten compounds showed very good binding affinity with the target protein. The retention time, compound name, molecular formula, molecular weight, percent area and the PubChem ID of these 10 identified phytocompounds against breast cancer target protein are shown in Table 1. The GC–MS chromatogram of the aqueous extract of *S. torvum* unripe fruits is shown in Fig. 1a,b.

**Table 1:** The phytocompounds identified from GC–MS analysis of the aqueous extract of *S. torvum* unripe fruits against breast cancer

S. no	RT	Compound name	Molecular formula	Molecular weight (g/mol)	Percent area	PubChem (CID)
1	23.065	Ergost-25-ene-3,6-dione, 5,12-dihydroxy-, (5.alpha.,12.beta.)-	C_28_H_44_O_4_	444.6	0.26	91,692,405
2	17.722	Aspidospermidin-17-Ol, 1-acetyl-16-methoxy-	C_22_H_30_N_2_O_3_	370.5	0.45	632,854
3	15.286	2-[3,4-dichlorophenyl]-4-[[2-[1-methyl-2-pyrrolidinyl]ethyl]amino]-6-[trichloromethyl]-S-triazine	C_17_H_18_C_l5_N_5_	467	1.10	558,706
4	46.491	Dihydroartemisinin, 10-O-(T-butyloxy)-	C_19_H_3_2O_6_	356.5	0.07	537,898
5	43.664	3-[(2-fluoroanilino)methyl]-5-(2-methoxyphenyl)-1,3,4-oxadiazole-2(3 h)-thione	C_16_H_14_FN_3_O_2_S	331.4	0.27	578,971
6	35.204	2(1H)-naphthalenone, 5-[2-(3-furanyl)ethyl]octahydro-1,5,6,8a-tetramethyl-,	C_20_H_30_O_2_	302.5	0.01	565,269
7	43.270	4H-1-benzopyran-4-one, 2-(3,4-dimethoxyphenyl)-3,5-dihydroxy-7-methoxy-	C_18_H_16_O_7_	344.3	0.31	5,748,558
8	28.017	2-butenoic acid, 2-methyl-, 4,4a,5,6,7,8,8a,9-octahydro-8a-hydroxy-3,4a,5-trimethylnaphtho[2,3-B]furan-6-yl ester	C_20_H_28_O_4_	332.4	0.14	5,367,763
9	18.747	Benzonitrile, 4-(4-ethylcyclohexyl)-, trans-	C_15_H_19_N	213.32	0.27	175,307
10	31.404	1-(2-adamantylidene)semicarbazide	C_11_H_17_N_3_O	207.27	0.04	541,478

From the results (Table 1), the best compounds ergost-25-ene-3,6-dione,5,12-dihydroxy-,(5.alpha.,12.beta.), aspidospermidin-17-ol,1-acetyl-16-methoxy and 2-(3,4-dichlorophenyl)-4-[[2-[1-methyl-2-pyrrolidinyl]ethyl amino]-6-[trichloromethyl]-S-triazine were observed in the retention time of 23.065 (0.26%), 17.722 (0.45%) and 15.286 (1.10%), respectively.

The 2D structure of the best identified phytocompounds from GC–MS analysis is shown in Table 2, and the GC–MS chromatogram of the best phytocompounds is shown in Figs. 2, 3, 4, 5, 6, 7, 8, 9, 10 and 11.

**Table 2:**
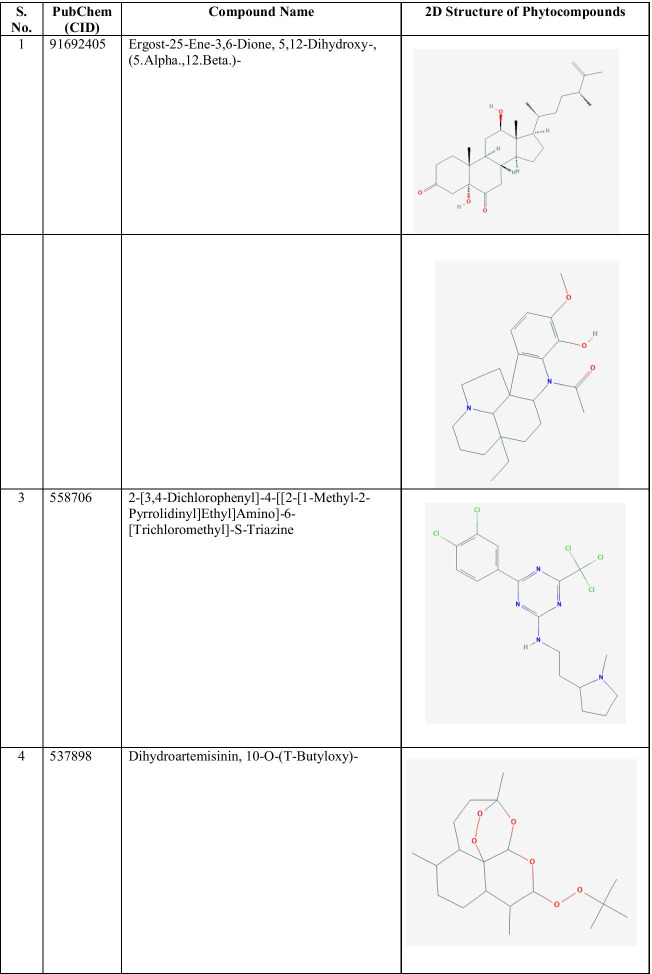

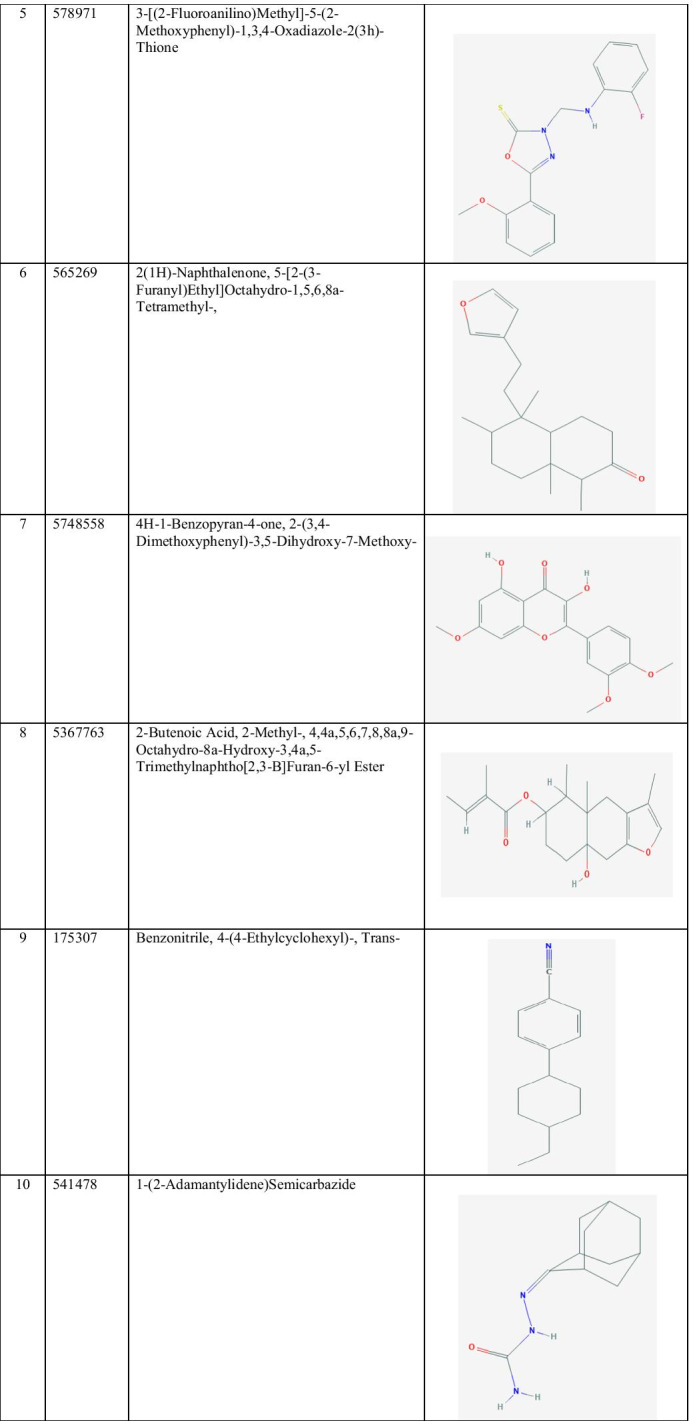
The 2D structure of the best identified phytocompounds from GC–MS analysis

**Fig. 2:**
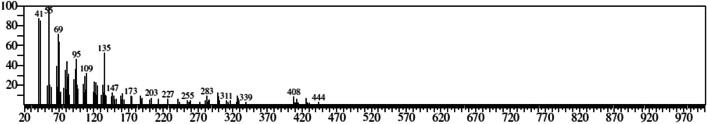
GC–MS chromatogram of ergost-25-ene-3,6-dione, 5,12-dihydroxy-, (5.alpha.,12.beta.)-

**Fig. 3:**
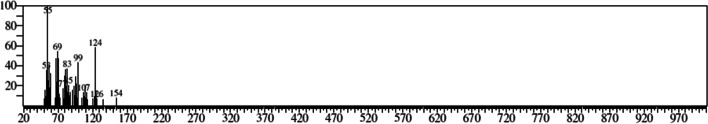
GC–MS chromatogram of aspidospermidin-17-ol, 1-acetyl-16-methoxy-

**Fig. 4:**
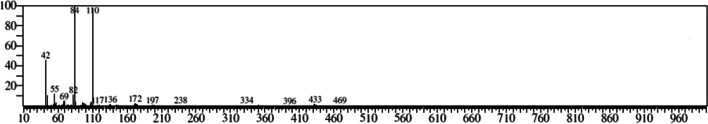
GC–MS chromatogram of 2-[3,4-dichlorophenyl]-4-[[2-[1-methyl-2-pyrrolidinyl]ethyl]amino]-6-[trichloromethyl]-S-triazine

**Fig. 5:**
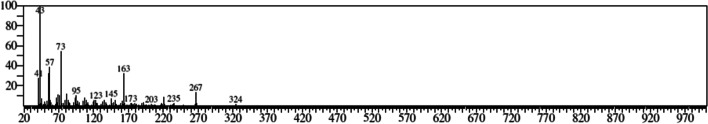
GC–MS chromatogram of dihydroartemisinin, 10-O-(t-butyloxy)-

**Fig. 6:**
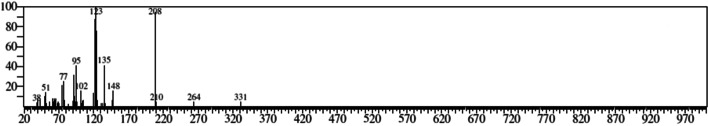
GC–MS chromatogram of 3-[(2-fluoroanilino)methyl]-5-(2-methoxyphenyl)-1,3,4-oxadiazole-2(3 h)-thione

**Fig. 7:**
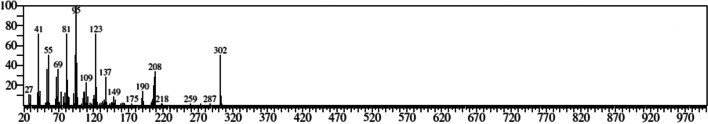
GC–MS chromatogram of 2(1H)-naphthalenone, 5-[2-(3-furanyl)ethyl]octahydro-1,5,6,8a-tetramethyl-,

**Fig. 8:**
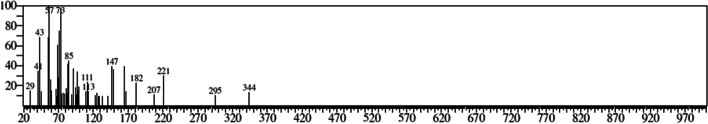
GC–MS chromatogram of 4H-1-benzopyran-4-one, 2-(3,4-dimethoxyphenyl)-3,5-dihydroxy-7-methoxy-

**Fig. 9:**
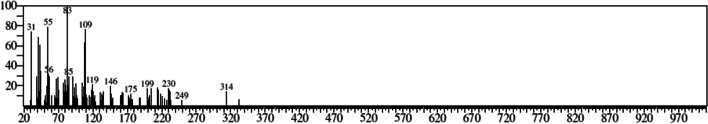
GC–MS chromatogram of 2-butenoic acid, 2-methyl-, 4,4a,5,6,7,8,8a,9-octahydro-8a-hydroxy-3,4a,5-trimethylnaphtho[2,3-b]furan-6-yl ester

**Fig. 10:**
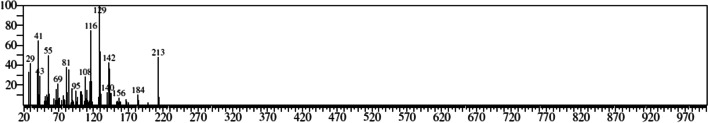
GC–MS chromatogram of benzonitrile, 4-(4-ethylcyclohexyl)-, trans-

**Fig. 11:**
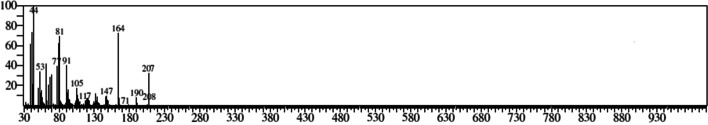
GC–MS chromatogram of 1-(2-adamantylidene) semicarbazide

### Docking Studies

**Fig. 12:**
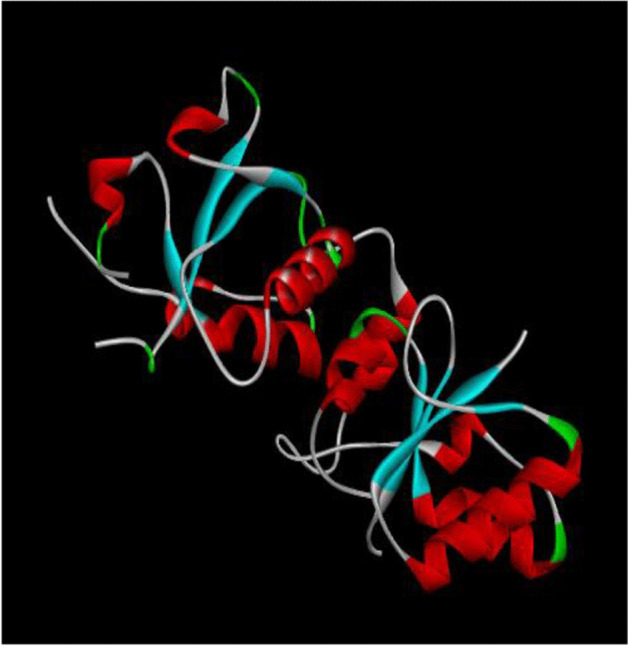
The 3D structure of target protein breast cancer type 1 susceptibility protein

The 3D structure of target protein breast cancer type 1 susceptibility protein is depicted in Fig. 12. A total of 236 phytocompounds were identified from GC–MS analysis of the aqueous extract of *S. torvum* unripe fruits, in which, 165 compounds were docked with breast cancer target protein. Among these, 10 compounds showed good binding affinity with the target protein. Further, the synthetic drug doxorubicin was also docked to find the interaction and binding affinity with the target protein. The results of the docking studies with the total number of bonds, interacting amino acid residues of the target protein and the bond length are presented in Table 3.

**Table 3:** Interaction of phytocompounds from the aqueous extract of *S. torvum* unripe fruits with the target protein breast cancer type 1 susceptibility protein

S. no	Compound name	Binding affinity (kcal/mol)	No. of bonds	Interacting residues	Bond length (Å)
1	Ergost-25-ene-3,6-dione, 5,12-dihydroxy-, (5.alpha.,12.beta.)-			TYR 1666	4.01
		− 7.3	4	MET 1663 GLY 1656 LEU 1657	5.23 2.38 2.49
2	Aspidospermidin-17-Ol, 1-acetyl-16-methoxy-			PRO 1776	4.74
		− 6.7	7	PRO 1776 LEU 1701 LEU 1701 LYS 1702 LYS 1702 LYS 1702	5.27 4.98 4.93 3.96 4.05 2.33
3	2-[3,4-dichlorophenyl]-4-[[2-[1-methyl-2-pyrrolidinyl]ethyl]amino]-6-[trichloromethyl]-S-triazine			ILE 1680	2.22
		− 6.6	7	TRP 1782 TRP 1782 TRP 1782 TRP 1782 TRP 1782 TRP 1782	3.76 3.91 4.19 5.38 3.97 5.00
4	Dihydroartemisinin, 10-O-(t-butyloxy)-			GLU 1836	3.31
		− 6.5	4	ARG 1835 ARG 1835 GLN 1811	2.51 2.59 2.10
5	3-[(2-fluoroanilino)methyl]-5-(2-methoxyphenyl)-1,3,4-oxadiazole-2(3 h)-thione			TRP 1782	3.81
		− 6.4	7	TRP 1782 GLN 1779 LEU 1701 LYS 1702 ILE 1680 LEU 1679	4.02 3.37 5.15 5.40 2.66 5.12
6	2(1H)-naphthalenone, 5-[2-(3-furanyl)ethyl]octahydro-1,5,6,8a-tetramethyl-,			LEU 1701	3.20
		− 6.3	3	LEU 1701 LYS 1702	4.80 5.40
7	4H-1-benzopyran-4-one, 2-(3,4-dimethoxyphenyl)-3,5-dihydroxy-7-methoxy-			THR 1799	2.41
		− 6.3	9	THR 1802 THR 1802 PRO 1806 PRO 1806 GLU 1829 CYS 1828 CYS 1828 LEU 1795	2.73 3.57 4.19 4.83 3.68 4.80 5.42 3.60
8	2-butenoic acid, 2-methyl-, 4,4a,5,6,7,8,8a,9-octahydro-8a-hydroxy-3,4a,5-trimethylnaphtho[2,3-b]furan-6-yl ester			PRO 1831	5.44
		− 6.2	4	THR 1799 MET 1827 CYS 1828	2.69 3.53 4.80
9	Benzonitrile, 4-(4-ethylcyclohexyl)-, trans-			PHE 1695	5.00
		− 5.8	3	PHE 1717 PHE 1717	3.94 5.17
10	1-(2-adamantylidene) semicarbazide			LEU 1854	4.42
		− 5.8	4	GLY 1825 CYS 1828 GLU 1829	2.55 2.68 2.21
Synthetic drug:					
				VAL 1740	4.79
11	Doxorubicin	− 7.2	11	VAL 1740 VAL 1741 ASP 1840 ASP 1840 ASP 1840 THR 1852 TYR 1853 TYR 1853 PRO 1812 GLN 1811	3.53 3.98 3.48 3.22 3.71 1.95 5.73 5.57 3.45 2.14

The phytocompound ergost-25-ene-3,6-dione, 5,12-dihydroxy-, (5.alpha.,12.beta.) showed very good binding affinity (− 7.3 kcal/mol) with the amino acid residues TYR 1666, MET 1663, and LEU 1657 of target protein. The lowest binding affinity of − 5.8 kcal/mol was observed between the phytocompound 1-(2-adamantylidene) semicarbazide and the amino acid residues such as LEU 1854, GLY 1825, CYS 1828, and GLU 1829 of target protein.

**Fig. 13:**
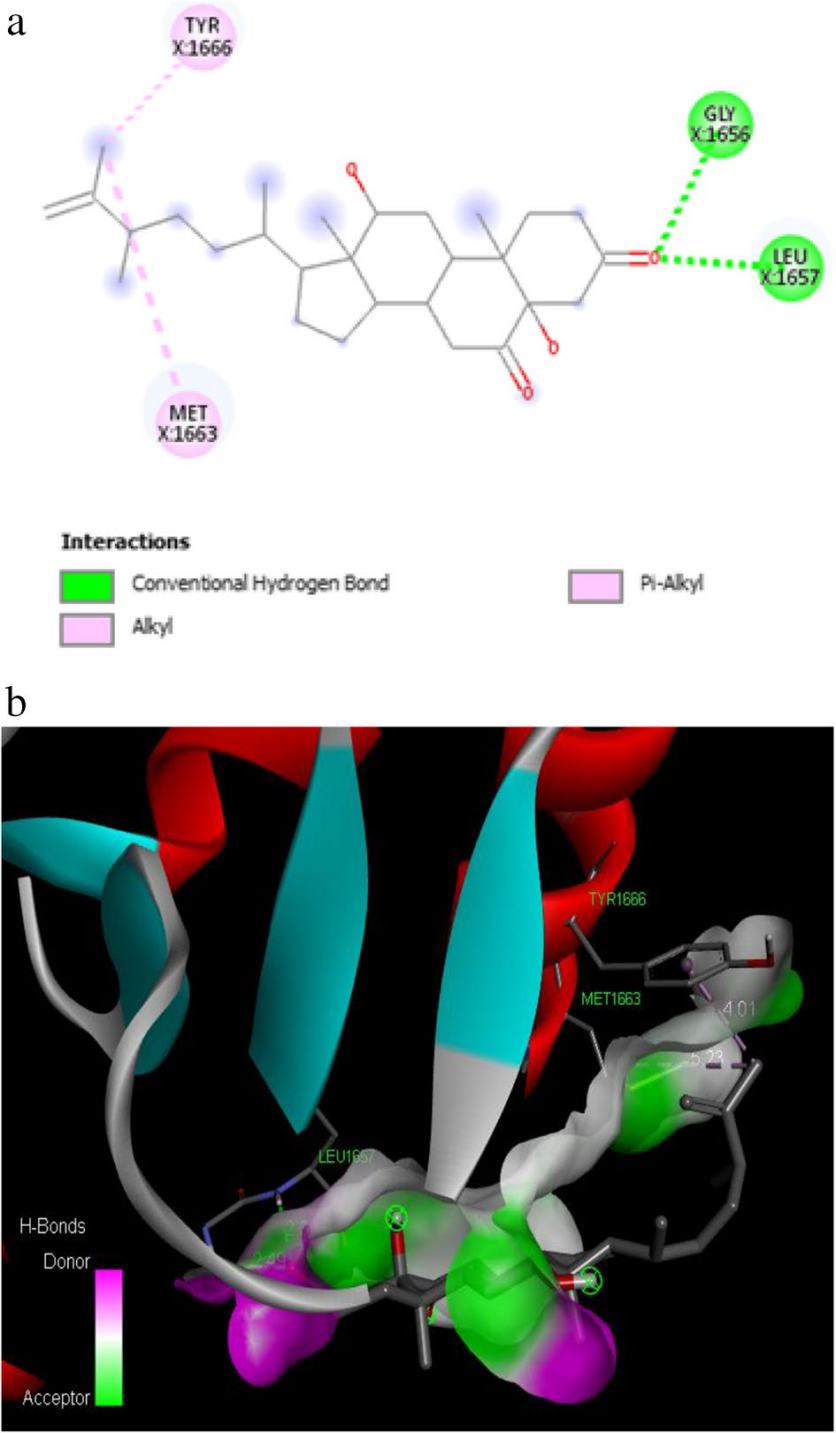
a 2D interaction of phytocompound ergost-25-ene-3,6-dione, 5,12-dihydroxy-, (5.alpha.,12.beta.) with the target protein. b 3D interaction of phytocompound ergost-25-ene-3,6-dione, 5,12-dihydroxy-, (5.alpha.,12.beta.) with the target protein

**Fig. 14:**
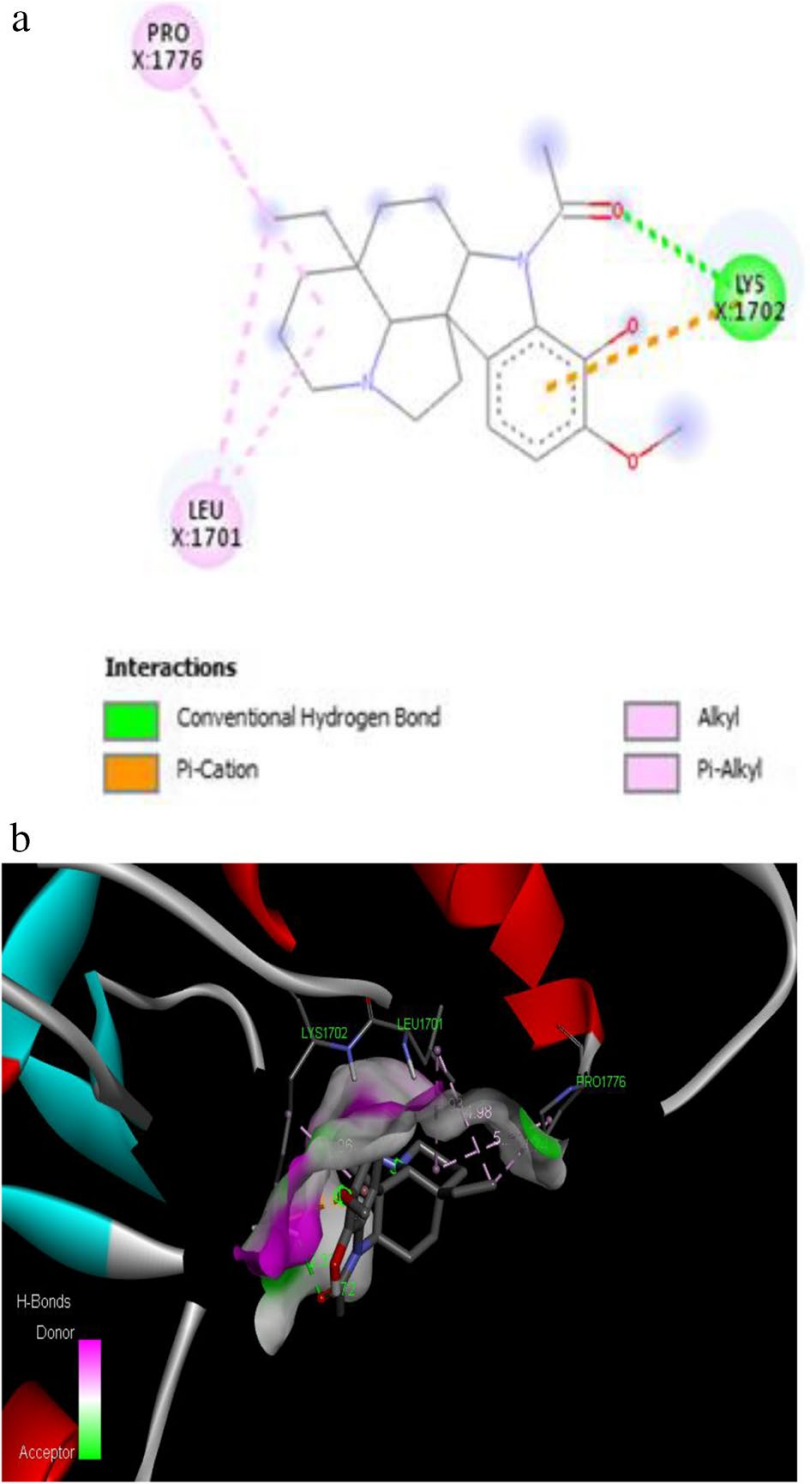
a 2D interaction of phytocompound aspidospermidin-17-Ol, 1-acetyl-16-methoxy- with the target protein. b 3D interaction of phytocompound aspidospermidin-17-Ol, 1-acetyl-16-methoxy- with the target protein

**Fig. 15:**
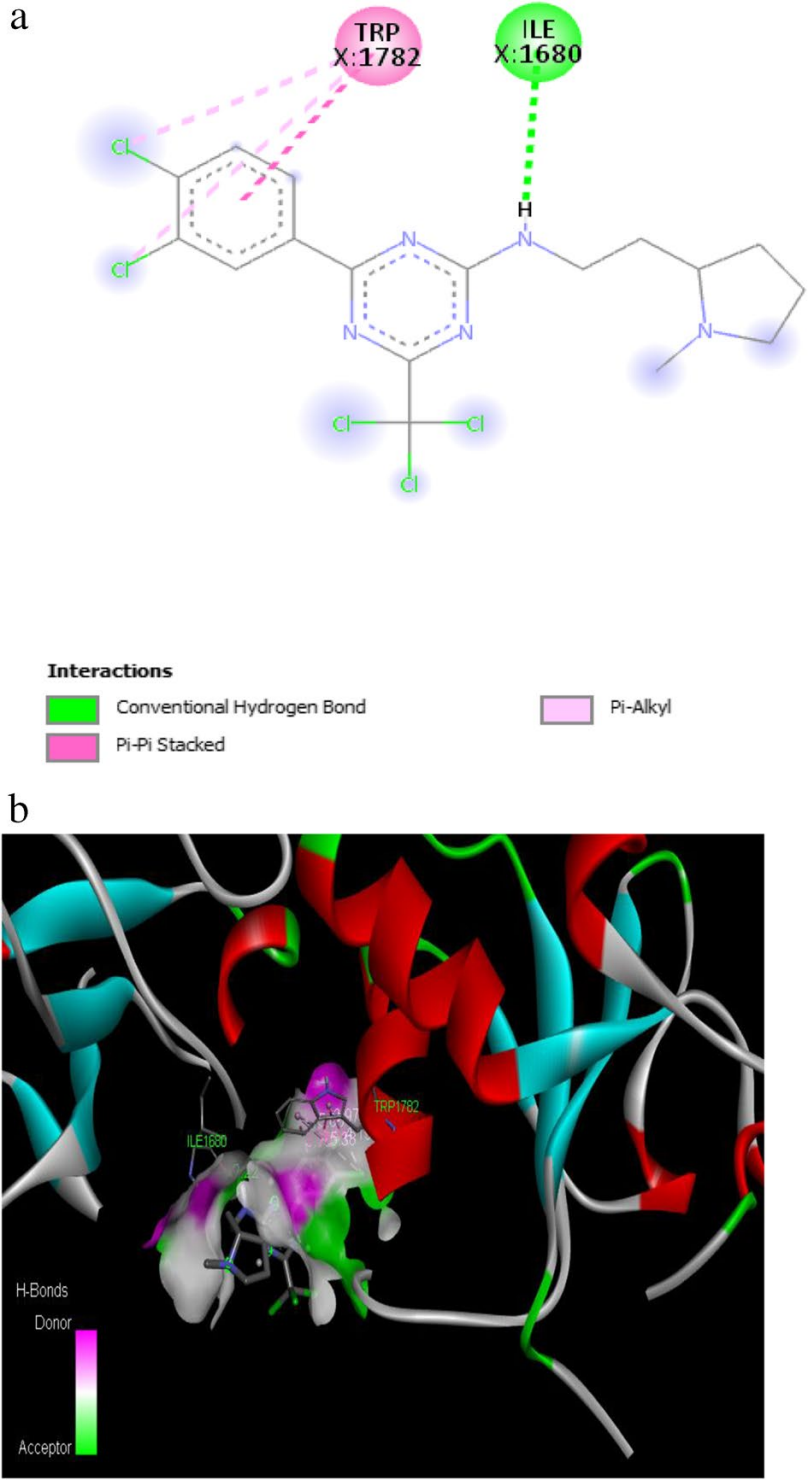
a 2D interaction of phytocompound 2-[3,4-dichlorophenyl]-4-[[2-[1-methyl-2-pyrrolidinyl]ethyl]amino]-6-[trichloromethyl]-S-triazine with the target protein. b 3D interaction of phytocompound 2-[3,4-dichlorophenyl]-4-[[2-[1-methyl-2-pyrrolidinyl]ethyl]amino]-6-[trichloromethyl]-S-triazine with the target protein

**Fig. 16:**
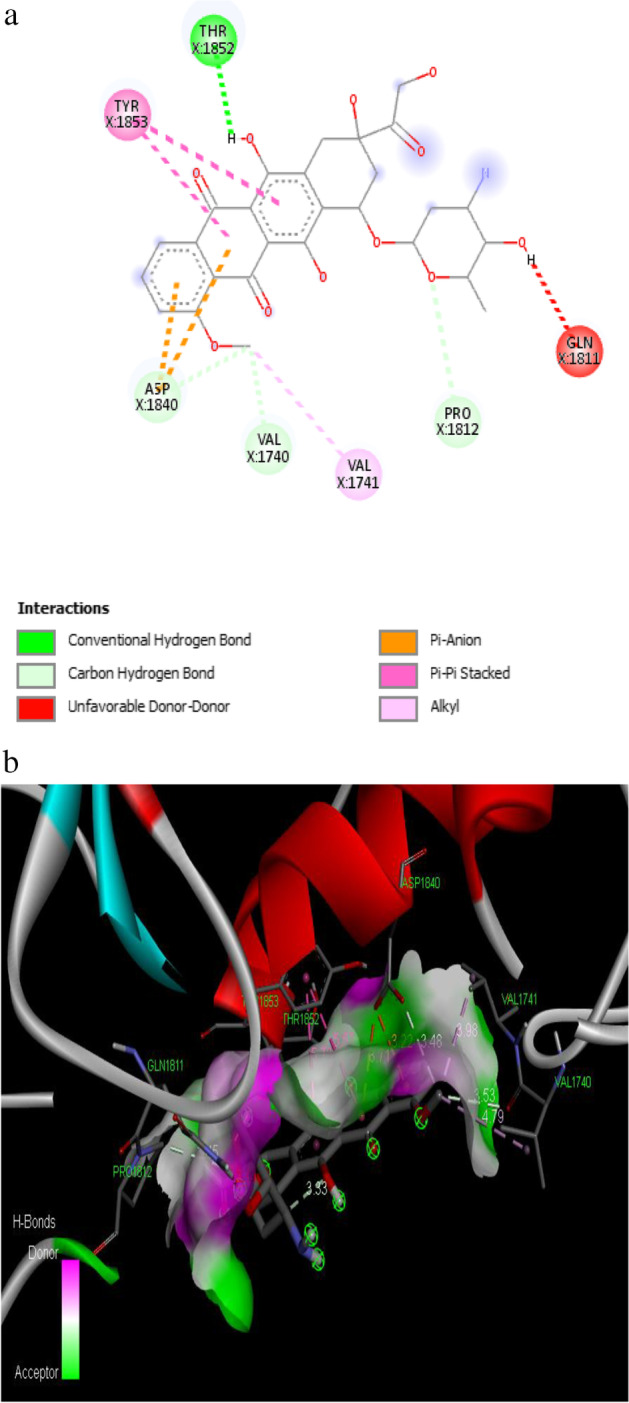
a 2D interaction of synthetic drug doxorubicin with the target protein. b 3D interaction of synthetic drug doxorubicin with the target protein

The binding affinity of − 7.2 kcal/mol was reported for the synthetic drug doxorubicin and the target protein with the amino acid residues of VAL 1740, VAL 1741, ASP 1840, THR 1852, TYR 1853, PRO 1812 and GLN 1811. The 2D and 3D interaction of the first three best interacted phytocompounds (ligands) and synthetic drug doxorubicin with the target protein is depicted in Figs. 13, 14, 15 and 16.

### Physicochemical and Pharmacokinetic Properties of Phytocompounds

ADMET physicochemical and pharmacokinetic properties were tested for the ten best interacted phytocompounds of the aqueous extract of *S. torvum* unripe fruits and synthetic drug doxorubicin.

The compounds identified exhibited molecular weight ranging less than 500 g/mol, while for doxorubicin it was 543.5 g/mol. The X Log P_3_ value of 7 compounds was between the ranges of 1.00 and 5.82. The TPSA value of the compounds was within the range of 23.79 to 98.36. Log S values ranged from − 1.62 to − 5.67. Fraction C sp3 values were in the range of 0.12 to 1.00. Rotatable bonds of all the compounds were within the limit between 2 and 6 (Table 4).

**Table 4:** Physicochemical properties of identified compounds from the aqueous extract of *S. torvum* unripe fruits

Compound no	Compound name	Molecular weight (g/mol)	X log P3	TPSA (Å)	Log S (ESOL)	Fraction Csp3	Rotatable bonds
1	Ergost-25-ene-3,6-dione, 5,12-dihydroxy-, (5.alpha.,12.beta.)-	444.6	5.40	74.60	− 5.67	0.86	5
2	Aspidospermidin-17-Ol, 1-acetyl-16-methoxy-	370.5	3.02	53.01	− 4.01	0.68	3
3	2-[3,4-dichlorophenyl]-4-[[2-[1-methyl-2-pyrrolidinyl]ethyl]amino]-6-[trichloromethyl]-S-triazine	467	5.82	53.94	− 6.35	0.47	6
4	Dihydroartemisinin, 10-O-(t-butyloxy)-	356.5	4.56	55.38	− 4.72	1.00	3
5	3-[(2-fluoroanilino)methyl]-5-(2-methoxyphenyl)-1,3,4-oxadiazole-2(3 h)-thione	331.4	4.06	84.31	− 4.67	0.12	5
6	2(1H)-naphthalenone, 5-[2-(3-furanyl)ethyl]octahydro-1,5,6,8a-tetramethyl-,	302.5	5.38	30.21	− 5.07	0.75	3
7	4H-1-benzopyran-4-one, 2-(3,4-dimethoxyphenyl)-3,5-dihydroxy-7-methoxy-	344.3	2.52	98.36	− 3.77	0.17	4
8	2-butenoic acid, 2-methyl-, 4,4a,5,6,7,8,8a,9-octahydro-8a-hydroxy-3,4a,5-trimethylnaphtho[2,3-b]furan-6-yl ester	332.4	3.97	59.67	− 4.36	0.65	3
9	Benzonitrile, 4-(4-ethylcyclohexyl)-, trans-	213.32	4.97	23.79	− 4.44	0.53	2
10	1-(2-adamantylidene) semicarbazide	207.27	1.00	67.48	− 1.62	0.82	2
11	Doxorubicin (synthetic drug)	543.5	1.27	206.07	− 3.91	0.44	5

The phytocompounds that best interacted obey Lipinski rule of five, while the synthetic drug doxorubicin did not obey Lipinski rule. Most of the compounds cross BBB and had high intestinal absorption. A bioavailability score of 0.55 was observed in the phytocompounds analyzed, and for doxorubicin it was 0.17 (Table 5).

**Table 5:** Pharmacokinetic properties of identified compounds from the aqueous extract of *S. torvum* unripe fruits

Compound no	Compound name	Lipinski	BBB	HIA	PGP-	Bioavailability score
1	Ergost-25-ene-3,6-dione, 5,12-dihydroxy-, (5.alpha.,12.beta.)-	Yes	No	High	No	0.55
2	Aspidospermidin-17-Ol, 1-Acetyl-16-Methoxy-	Yes	Yes	High	Yes	0.55
3	2-[3,4-Dichlorophenyl]-4-[[2-[1-methyl-2-pyrrolidinyl]ethyl]amino]-6-[trichloromethyl]-S-triazine	Yes	Yes	High	Yes	0.55
4	Dihydroartemisinin, 10-O-(t-butyloxy)-	Yes	Yes	High	Yes	0.55
5	3-[(2-fluoroanilino)methyl]-5-(2-methoxyphenyl)-1,3,4-oxadiazole-2(3 h)-thione	Yes	No	High	Yes	0.55
6	2(1H)-naphthalenone, 5-[2-(3-furanyl)ethyl]octahydro-1,5,6,8a-tetramethyl-,	Yes	Yes	High	Yes	0.55
7	4H-1-benzopyran-4-one, 2-(3,4-dimethoxyphenyl)-3,5-dihydroxy-7-methoxy-	Yes	No	High	Yes	0.55
8	2-butenoic acid, 2-methyl-, 4,4a,5,6,7,8,8a,9-octahydro-8a-hydroxy-3,4a,5-trimethylnaphtho[2,3-b]furan-6-yl ester	Yes	Yes	High	Yes	0.55
9	Benzonitrile, 4-(4-ethylcyclohexyl)-, trans-	Yes	Yes	High	Yes	0.55
10	1-(2-adamantylidene) semicarbazide	Yes	Yes	High	No	0.55
11	Doxorubicin (synthetic drug)	No	No	Low	NA	0.17

The compounds ergost-25-ene-3,6-dione, 5,12-dihydroxy-, (5.alpha.,12.beta.) and 1-(2-adamantylidene) semicarbazide did not inhibit any CYP450 enzymes. The compounds aspidospermidin-17-Ol, 1-acetyl-16-methoxy and dihydroartemisinin, 10-O-(t-butyloxy) inhibited one CYP450 enzyme, CYP2D6 and CYP1A2, respectively. The compounds 2(1H)-naphthalenone, 5-[2-(3-furanyl)ethyl]octahydro-1,5,6,8a-tetramethyl and 2-butenoic acid, 2-methyl-, 4,4a,5,6,7,8,8a,9-octahydro-8a-hydroxy-3,4a,5-trimethylnaphtho[2,3-b]furan-6-yl ester inhibited the CYP450 enzyme, CYP2D6.

The phytocompounds 2-[3,4-dichlorophenyl]-4-[[2-[1-methyl-2-pyrrolidinyl]ethyl]amino]-6-[trichloromethyl]-S-triazine, 3-[(2-fluoroanilino)methyl]-5-(2-methoxyphenyl)-1,3,4-oxadiazole-2(3H)-thione and 4H-1-benzopyran-4-one, 2-(3,4-dimethoxyphenyl)-3,5-dihydroxy-7-methoxy inhibited 4 CYP450 enzymes.

The compound having a high negative value (log *K*_p_) has less skin permeation ability The compounds aspidospermidin- 17-0 l, 1-acetyl-16-methoxy, 4H-1-benzopyran-4-one,2-(3,4-dimethoxyphenyl)-3,5-dihydroxy-7-methoxy and 1-(2-adamantylidene) semicarbazide have less skin permeation ability. The cytochrome and skin permeation properties are tabulated (Table 6).

**Table 6:** Cytochrome properties and skin permeation of identified compounds from the aqueous extract of *S. torvum* unripe fruits

Compound no	Compound name	CYP1A2 inhibitor	CYP2C19 inhibitor	CYP2C9 inhibitor	CYP2D6 inhibitor	CYP3A4 inhibitor	Log *K*_p_ (Skin permeation) (cm/s)
1	Ergost-25-ene-3,6-dione, 5,12-dihydroxy-, (5.alpha.,12.beta.)-	No	No	No	No	No	− 5.18
2	Aspidospermidin-17-Ol, 1-acetyl-16-methoxy-	No	No	No	Yes	No	− 6.42
3	2-[3,4-dichlorophenyl]-4-[[2-[1-methyl-2-pyrrolidinyl]ethyl]amino]-6-[trichloromethyl]-S-triazine	No	Yes	Yes	Yes	Yes	− 5.03
4	Dihydroartemisinin, 10-O-(t-butyloxy)-	Yes	No	No	No	No	− 5.24
5	3-[(2-fluoroanilino)methyl]-5-(2-methoxyphenyl)-1,3,4-oxadiazole-2(3 h)-thione	Yes	Yes	Yes	No	Yes	− 5.44
6	2(1H)-naphthalenone, 5-[2-(3-furanyl)ethyl]octahydro-1,5,6,8a-tetramethyl-,	No	No	No	Yes	No	− 4.33
7	4H-1-benzopyran-4-one, 2-(3,4-dimethoxyphenyl)-3,5-dihydroxy-7-methoxy-	Yes	No	Yes	Yes	Yes	− 6.61
8	2-butenoic acid, 2-methyl-, 4,4a,5,6,7,8,8a,9-octahydro-8a-hydroxy-3,4a,5-trimethylnaphtho[2,3-b]furan-6-yl ester	No	No	No	Yes	No	− 5.51
9	Benzonitrile, 4-(4-ethylcyclohexyl)-, trans-	No	No	Yes	Yes	No	− 4.07
10	1-(2-adamantylidene) semicarbazide	No	No	No	No	No	− 6.85
11	Doxorubicin (synthetic drug)	No	No	No	No	No	− 8.71

## Discussion

Natural products are gaining importance in the discovery of anticancer antioxidant-based lead molecules for cancer treatment [33, 34]. Computational algorithm methods are well documented in medicinal synthetic chemistry; however, their application in the field of natural phytocompounds remains scanty and unexplored. The aim of molecular docking contributes to the prediction of the ligand-receptor complex structure by computational approaches [35]. Docking mechanism executes virtual identification on library store of compounds; the results are aligned based on the scores, and structural hypothetical theories are formulated on how the ligands inhibit the target receptor, which is vital in lead enhancement [36]. The various factors affecting docking are the intramolecular forces like bond width, bond angle and dihedral angle and intermolecular forces which include electrostatic, dipolar, hydrogen bonding and hydrophobicity [37, 38].

Molecular docking studies were done for the phytocompounds of *S. torvum* unripe fruits with BRCA1 protein. BRCA1 plays a vital role in DNA repair, transcriptional regulation and tumor suppressor functions. The phytochemicals and commercial drug doxorubicin were docked with the active site of the target protein and help to enhance its function. However, as the commercial drug doxorubicin did not obey the Lipinski rule of five and the identified phytocompounds in our study obey the Lipinski rule of five, the present study concludes that the commercial drug doxorubicin is toxic and the identified phytocompounds in our study are non-toxic to humans. The binding affinity, different types of bonds specifically hydrogen bonds and interaction of amino acid residues with the ligand, bond length between the atom of ligand and target protein were observed. The binding affinities of the target proteins were obtained for all the phytocompounds (ligands) in terms of kcal/mol [39]. The residual interaction in the present study showed where the ligand exactly binds to particular amino acid of the protein. Out of 165 compounds docked, 10 compounds showed good binding affinity. Compounds 1, 2, 3, 4 and 5 reported binding affinity of − 7.3, − 6.7, − 6.6, − 6.5 and − 6.4 kcal/mol, respectively. The hydrogen bond indicates that the ligand had high binding affinity with the protein, and a high negative score indicates good binding affinity with the target protein [40, 41]. The binding affinity of the phytocompounds from *S. torvum* unripe fruits is close with the synthetic drug doxorubicin.

The important physicochemical properties, which are molecular weight, lipophilicity, polarity, solubility, saturation of carbon fractions and flexibility, represented by rotatable bonds are essential for the compounds to prove its drug-likeness [42]. Compounds which were identified with molecular weight less than or equal to 500 g/mol have the potential to be easily absorbed, diffused and transported. Lipophilic property influences the solubility, selectivity and permeability of possible drug-like compounds [43]. (XlogP_3_) values between the range − 0.7 and + 5.0 is proven to be a satisfying lead molecule. Compounds 1 and 6 show a very slight deviation whereas all other compounds do not deviate the range. High lipophilic nature of the compound leads to a higher rapid metabolic turnover, low solubility, negligible absorption in the intestine region and causing toxic effects to vital organs [44]. The polarity of the compounds between 20 and 130 Å, solubility not higher than 6 fits well within the acceptable range for drug-likeness. (Log S scale: insoluble <  − 10 < poorly <  − 6 < moderately <  − 4 < soluble <  − 2 < very soluble < 0 < highly soluble): The aqueous solubility is directly estimated from the compound’s molecular structure and molecular weight. Fraction of carbons in the sp3 hybridization not less than 0.25 prove to be efficient. Values of compounds 5 and 7 in the present study were less than 0.25, and the value of other compounds was above this limit. The rotatable bonds of not more than 9 determine the flexibility of the compound. The present study showed all the compounds with less than 9 rotatable bonds.

Lipinski formulated “Rule of 5” (Ro5) properties with molecular description about the compounds. The rule is helpful in the drug designing process. The present study states that the phytocompounds studied were found to be within the Lipinski’s limit range without any violation. Ro5 was used as a filter to identify compounds that have high probability of being drug candidates [45].

The gastrointestinal absorption of the identified compounds from the unripe fruit extract of *S. torvum* revealed the potential of being well absorbed in the gastrointestinal tract. It is suggested that these compounds are permeable from the gastrointestinal tract when they are orally administrated [46]. The BBB is a physiological barrier made up of microvascular endothelial cell layer of the brain which separates it from the blood stream. The phytocompounds were assessed for their ability to cross BBB. Eight percent of the compounds exhibit capability to cross the BBB. The penetration across BBB is a criteria for compounds targeting the central nervous system. Compounds 1, 5 and 7 did not show potential to cross BBB. This could be considered for exerting lesser adverse effects in the region of the central nervous system, whereas all other compounds had probabilities of crossing the BBB [47].

P-glycoproteins (P-gp) are compounds that act as membrane transporters in the intracellular or extracellular regions of the cell. P-glycoprotein plays a significant role in drug absorption and excretion [48]. Compound 2 to 9 are non-substrates for P-gp. This implies that the compounds if they are not a P-gp substrate would not be affected by the efflux action of P-gp, which in turn eliminates compounds from cells. Thus, only the efficacy of compound 1 and 10 has potential to be resisted in different target sites. This membrane transporter protein appears to have an impact on limiting cellular uptake of drugs from blood circulation into brain, from intestinal lumen into epithelial cells rather than on increasing the excretion of drugs from hepatic cells and renal tubules [49, 50].

All the compounds have the bioavailability value of 0.55 which implies that the compounds adhere to Lipinski rule of five and have 55% probabilities of being bioavailable. The bioavailability of drug taken orally is the fraction of the dose that reaches the bloodstream which is the crucial factor in drug designing. The bioavailability of a drug is determined mainly by gastrointestinal absorption. A drug should have good aqueous solubility for oral bioavailability and absorption [51, 52].

Cytochrome P450 monooxygenase enzyme plays an integrative role in drug metabolism and its elimination in biological systems. About 80% of the molecules in the present study identified are substrates of five isoforms CYP1A2, CYP2C19, CYP2C9, CYP2D6 and CYP3A4 [53]. The non-inhibitory action of the identified compounds against these enzymes indicated that these compounds have high probabilities of being transformed and consequently being bioavailable upon oral administration. These compounds do not inhibit the CYP450 enzymes and do not give any adverse reactions. The inhibition of the CYP isomers by these compounds can cause poor bioavailability due to metabolic derangements and toxic side effects due to their accumulation [54]. A few compounds in the present study inhibit the CYP450 enzymes and give unanticipated adverse reactions. Inhibition of these isoenzymes is a major concern of pharmacokinetics-related drug-drug interactions and its accumulation leading to toxic ADME of the drug and its metabolites [55].

The skin is a selective barrier that paves way for different compounds for its penetration. The skin permeability is a vital parameter for the assessment of compounds that might require transdermal administration. The more negative the log *K*_p_, the less skin permeability of the molecule. All the compounds in the present study are impermeable as they are represented with the negative log *K*_p_ values [56].

Hence, the present study concludes that the phytocompounds identified from the aqueous extract of *S. torvum* unripe fruits using GC–MS analysis suggest the potential ability to treat breast cancer. The first six compounds indicate good binding affinity when compared with the synthetic drug doxorubicin. ADMET parameters of lead compounds using computational assessments are adapted as they circumvent the high costs, prevent unnecessary use of resources and save time. Computational methods, though not confirmatory, do provide valuable information of the most likely drug-like compounds out of an array of identified compounds [57, 58].

Ro5 is used as a tool to identify compounds that have maximum probability for being considered as a potential drug. Drugs are to be easily absorbed, metabolized and eliminated from blood stream without causing any toxic effects [59]. These drugs are distributed to the targeted site of action in the body to interact with receptor molecules. These conditions envisage that these compounds are promising therapeutic alternatives to treat metabolic and degenerative disorders. The pharmacokinetic properties analyzed were fitted well within the acceptable range for human use [60].

## Data Availability

All data generated or analyzed during this study are included in this article.
